# Mental Healthcare workers’ experiences in managing psychiatric patients’ aggression in Maseru

**DOI:** 10.4102/hsag.v28i0.2324

**Published:** 2023-10-27

**Authors:** Libuseng M. Rathobei, Sipho W. Mkhize

**Affiliations:** 1 School of Nursing and Public Health, Faculty of Health Sciences, University of KwaZulu Natal, Durban, South Africa

**Keywords:** mental healthcare workers, management strategies, psychiatric patients, clinical facility, aggressive and violent behavior

## Abstract

**Background:**

Aggression and violence in psychiatric clinical facilities are a common case, and mental healthcare workers utilise various management strategies to combat it. It is therefore crucial for mental healthcare workers to acquire skills for the management strategies of aggressive behaviour of psychiatric patients.

**Aim:**

The aim of the study was to describe mental healthcare workers’ experiences in the management strategies of aggressive and violent behaviour by psychiatric patients in Maseru district.

**Setting:**

A psychiatric hospital situated to the south in Maseru in the rural countryside and general hospital located in the western lowlands of Lesotho in the village of Morija were used.

**Methods:**

This study adopted a qualitative, exploratory, descriptive and contextual research design. There were two focus group interviews conducted per clinical facility, which consisted of six members in each group. There were four participants for individual interviews from psychiatric clinical facility and three individual interviews from general clinical facility.

**Results:**

Thematic analysis of the data resulted in themes and sub-themes. These were psychological intervention strategy, physical strategy, chemical strategy, empowerment strategy and policy strategy. Participants viewed various management strategies of aggressive and violent behaviour of psychiatric patients they utilise in clinical facilities as effective.

**Conclusion:**

The mental healthcare workers’ experiences in managing aggression and violent behaviour of psychiatric patients were expression of psychological, physical, chemical, empowerment and policy strategies.

**Contribution:**

The study will enhance the knowledge, skills and attitudes regarding management strategies that mental healthcare workers can utilise in managing aggressive and violent behaviour of psychiatric patients.

## Introduction

Aggression in psychiatric clinical facilities is a common occurrence that mental healthcare workers have difficulty in managing (Njaka et al. 2020). Tomagová et al. (2016) stated that mental healthcare workers utilise various management strategies of aggressive and violent behaviour by psychiatric patients. Mental healthcare worker is any person providing mental healthcare services to psychiatric patients (Uys & Middleton 2015). However, Ayhan and Hicdurmaz (2020) indicated that inappropriate interventions intended to de-escalate patient’s aggression can jeopardise the quality of patient care. This article describes the mental healthcare workers’ experience in the management strategies of aggressive and violent behaviour by psychiatric patients in the Maseru district.

The National Institute for Health and Care Excellence (NICE) guidelines (2015) define aggression as a range of behaviours or actions that can result in harm, hurt or injury to another person, regardless of whether the expression of violence or aggression is physical or verbal. Furthermore, Rathobei, Nyangu and Dube (2021) stated that aggression is behaviour categorised by anger, hostile thoughts, words and actions towards other people that are noticeable in speech, tone of voice, body language, outward expression of anger or rage that is threatening and actual or physical actions. In addition, violence is the deliberate use of physical strength or power, threatened or actual, against oneself or against other people, which may result in injury, death, psychological harm, mal-development or deprivation, while aggression is an argumentative, injurious or destructive behaviour to self or others (Gillions et al. 2019). Pelto-Piri, Warg and Kjellin (2020) asserted that aggression is a serious problem in the psychiatric inpatient care environment affecting mental healthcare workers’ physical and psychological health. Therefore, mental healthcare workers experience a significant proportion of aggression and violence that negatively impacts their physical and psychological well-being, which ultimately limits their work performance and job satisfaction (Kumari et al. 2020). Consequently, Gadegaard, Andersen and Hogh (2018) indicated that aggression and violence exposures of mental healthcare workers warrant preventive action.

Ayhan and Hicdurmaz (2020) specified that alternative aggression and violence preventive methods include interventions such as clarifying the needs of patient through communication, calming the patient down and restructuring of the environment. Consequently, Baby, Gale and Swain (2018) asserted that communication is a skill required to build therapeutic relationships and a tool to deliver appropriate therapeutic interventions. Furthermore, Price et al. (2018) added that there needs to be empathy and respect conveyed and negative emotional responses inhibited when working with aggressive and violent patients. Ayhan and Hicdurmaz (2020) added that physical, chemical and mechanic restraints utilised in the management of aggression in psychiatric institutions are repressive and compelling methods and they have physical and emotional impacts on patients. Moreover, Lanthén, Rask and Sunnqvist (2015) reported that restraints can increase aggression and agitation in patients and cause negative effects on their behaviour in and after they leave hospital; therefore, interventions that reduce aggression without restrictive practices are a priority. However, Price et al. (2018) detailed that the restrictive practices are used to minimise harm from aggression and violence through restricting aggression and violent patient’s ability to act independently.

Aggression and violence guidelines emphasise that management should utilise ward policies to address aggression and violence through de-escalation techniques (Pelto-Piri et al. 2020). In addition, Goodman et al. (2020) reported that de-escalation techniques are a range of management strategies including non-physical or interpersonal approaches aimed to reduce violence and aggression and hence avoid the use of restrictive interventions by negotiating a mutually agreeable solution to the aggressor’s concerns. Consequently, Ayhan and Hicdurmaz (2020) indicated that perception of mental healthcare workers towards aggression determines their response and approach to aggressive and violent patients. It is, therefore, crucial for mental healthcare workers to engage in training for the management strategies of aggression and violence to equip themselves with knowledge, skills and confidence towards aggressive patients (Pelto-Piri et al. 2020). A study conducted in Turkey stated the majority of mental healthcare workers have not been trained in aggression and violence management (Arguvanli et al. 2015). In the study in the Slovak Republic, with 223 nurses, regarding the incidence rate of constituent forms of inpatient aggression towards nurses working in psychiatric inpatient wards, nurses expressed the strongest agreement with the use of medical therapy and restraints, and they held a neutral attitude towards the use of non-physical methods (Tomagová et al. 2016).

A study conducted in Nigeria revealed that nursing curriculum did not include aggression management but provides extensive information on the use of restraints (Oyelade & Mobolaji-Olajide 2019; Stockton, Vollm & Whittington 2015). Consequently, the study conducted in Egypt revealed that participants had confidence to deal with aggression after they had undergone the trainings (Abozaid et al. 2022). In the study conducted by Bekelepi, Martin and Chipps (2015) to determine the knowledge and skills of professional nurses in managing aggression of patients in psychiatric hospital, it was revealed that nurses had good (above 80) knowledge about management of aggressive psychiatric patients and that there is need for an ongoing in-service training and refresher course in managing aggression. However, information on mental healthcare workers’ experience in the management strategy of aggressive and violent behaviour by psychiatric patients, particularly in Maseru, remains limited. In this context, the current article seeks to explore mental healthcare workers’ experiences in the management strategy of aggressive and violent behaviour by psychiatric patients.

### Problem statement

The study conducted by Kumari et al. (2020) in Asia revealed that assessment of risk factors, development and implementation of workplace violence programmes and addressing underreporting of violent episodes have been suggested as some successful psychiatric mitigation strategies by participants. In addition, Bekelepi et al.’s (2015) study conducted in Western Cape, South Africa, revealed that all participants were aware of procedures to follow when reporting and documenting incidents of aggression.

In the context of Lesotho, however, there is no evidence-based research regarding mental healthcare workers’ experience concerning management strategies of aggressive and violent behaviour of psychiatric patients. According to the anecdotal evidence, mental healthcare workers utilise a variety of management strategies of aggressive and violent behaviours of psychiatric patients. It appears that mental healthcare workers need assistance in the management strategies of aggressive and violent psychiatric patients; hence, the proposed study seeks to explore mental healthcare workers’ experience in the management strategies of aggressive and violent behaviour of psychiatric patients in Maseru.

## Aim

To explore mental healthcare workers’ experiences in the management strategies of aggressive and violent behaviour of psychiatric patients in Maseru.

## Research design and methods

The study adopted a qualitative, explorative, descriptive, contextual research design. A constructivism paradigm was most suitable for this study because it was the most relevant avenue to explore and describe mental healthcare workers’ experiences in the management strategies of aggressive and violent behaviour.

### Participants

This research had 33 mental healthcare workers as participants. The participants’ ages ranged from 25 – 30 years old to 55 – 60 years old, and they worked in general (located in the western lowlands of Lesotho in the village of Morija, along the main road from the southern districts of the country to Maseru) and psychiatric (one referral psychiatric hospital situated to the south in Maseru district, in the rural countryside and next to a large army barracks) clinical facilities. The study recruited mental healthcare workers (psychiatric nurses, general nurses, medical doctors and nursing assistants) working in psychiatric and general clinical facilities with a psychiatric section. The sample selection was non-probability, purposive sampling and was obtained from participants who met the eligibility criteria. The criteria used for this study was the mental healthcare workers exposed to management strategies of aggressive behaviour of psychiatric patients in general and psychiatric clinical facilities in Lesotho. The selected participants in this study were ones exposed to management strategies of aggressive behaviour of psychiatric patients in general and psychiatric clinical facilities in Lesotho, who were representative of both genders, were healthcare workers working in selected general and psychiatric clinical facilities in Lesotho. The researcher predicted this group as a source of rich information that can be used to explain the phenomenon under study. Participant information can be found in Table 1 and Table 2.

**TABLE 1:** Individual interviews.

Gender	Participant number	Years of experience	Highest level of education
Male	1	25	Diploma in General Nursing & Midwifery
Male	2	2 years & 1 month	Bachelor Degree in Nursing & Midwifery
Male	3	11	Post Graduate Diploma in Psychiatric Nursing
Female	4	4	MBCHB
Female	5	10	Advanced Diploma in Nursing Management
Female	6	10	Post Graduate Diploma in Psychiatric Nursing
Female	7	22	Advanced Degree in Nursing Science
Male	8	6	Master’s Degree in Tropical Medicine
Female	9	4	Degree in Medicine

**TABLE 2:** Focus group interview.

Gender	Participant number	Years of experience	Highest level ofeducation
**Focus group interview 1**			
Female	A	8	Diploma in General Nursing & Midwifery
Female	B	8	Diploma in General Nursing & Midwifery
Female	C	9	Post Graduate Diploma in Nursing Management
Female	D	14	Bachelor in Nursing Science
Male	E	7	Diploma in Nursing & Midwifery
Male	F	5	Diploma in General Nursing & Midwifery
**Focus group interview 2**			
Male	01	5	Certificate in Nursing Assistant
Female	02	14	Certificate in Nursing Assistant
Female	03	20	Certificate in Nursing Assistant
Female	04	11	Certificate in Nursing Assistant
Male	05	16	Certificate in Nursing Assistant
Female	06	4	Certificate in Nursing Assistant
**Focus group interview 3**			
Male	A	33	Certificate in Nursing Assistant
Female	B	10	Certificate in Nursing Assistant
Female	C	17	Certificate in Nursing Assistant
Female	D	25	Certificate in Nursing Assistant
Female	E	33	Certificate in Nursing Assistant
Female	F	10	Certificate in Nursing Assistant
**Focus group interview 4**			
Male	A	10	Diploma in General Nursing & Midwifery
Female	B	3	Post Graduate Diploma in Psychiatric Nursing
Female	C	10	Post Graduate Diploma in Psychiatric Nursing
Male	D	18	Diploma in General Nursing & Midwifery
Male	E	12	Degree in General Nursing & Midwifery
Male	F	7	Post Graduate Diploma in Psychiatric Nursing

### Data collection

The interviews aimed to explore and describe the management strategies of aggressive behaviour of psychiatric patients. Two focus group interviews with six members in each clinical facility to draw upon participants’ attitudes, feelings, beliefs, experiences and interactions in a way in which would not be feasible using other methods were conducted and five individual interviews in psychiatric clinical facility and four individual interviews from general clinical facilities, which drew data about participant’s own experiences, used open-ended questions to elicit responses from mental healthcare workers. Participants who directly took care and who were always with the aggressive psychiatric patients participated in the focus group interviews, while in the individual interviews, all mental healthcare workers participated. Data collection was done for 5 months by the researcher in individual interview and by both researcher and research facilitator in focus group interviews. Interviews were conducted in English in a private space, in the academic block of the psychiatric hospital and also in the private unit that was adequate and lasted for about 45 min – 60 min. Questions included,

- What strategies do you use in the management of aggressive behaviour of psychiatric patients?
- Where do you use the management strategies of aggressive behaviour of psychiatric patients?
- How do you feel or react regarding the management strategies of aggressive behaviour of psychiatric patients?
- What do you know regarding the management strategies of aggressive behaviour of psychiatric patients?
- what do you think should be done regarding the management strategies of aggressive behaviour of psychiatric patients?

Grove et al. (2012) asserted that the researcher must comply with three principles as stated by the Belmont report, namely, the principle of beneficence, the principle of human dignity and justice. Beneficence seeks to do good or to benefit participants, so research should benefit individual participants and society as a whole (Brink et al. 2018). The participants were called using alphabetical letters. The participants were, therefore, provided with glass of water, tissue paper and counselling services for possible harm as they reveal their experiences regarding the management of aggressive behaviour of psychiatric patients. The principle of respect denotes that individuals are autonomous, that is, they have the right to self-determination (Brink et al. 2012). Participants were, therefore, given information regarding the research study and that they have the right to participate or not participate in the study, without any prejudices. If they agreed to participate in the study, they were given informed consent which they sign to show respect and autonomy. To respect the rights of participants, the researcher explained the purpose of the study to them and explained that participation in the study was voluntary and they had a right to withdraw at any time if they felt uncomfortable without fear of any negative effects. The researcher also explained that the participants will experience no harm by participating in the study. But for any emotional feelings the participants felt when answering questions during the interview, warm water, and a tissue paper were offered and the interview was held next to the professionals who providedpsychological help. After providing all the necessary information regarding the study, a signed consent form was obtained from those who voluntarily accepted to participate. Interviews were audio taped and transcribed in English by researcher at the end of each day of interviews. Interviews continued with participants until there was data saturation achieved and no new information uncovered.

### Data analysis

Data analysis happened shortly after data collection began. The researcher took field notes on all responses from participants. When assessing data, the Maguire and Delahunt (2017) theme analysis method was adopted, whereby the researcher gained familiarity with data through reading and re-reading transcripts of both the focus group and individual interviews. Then, the first codes were generated from each participant in focus group and also each participant in individual interviews. Coding, which lowers large amounts of data into small pieces of meaning, was then used to organise the data in a meaningful and systematic manner, with each segment of data that was related to or captured anything noteworthy about the study issue being coded. Each transcript was coded thematically based on every text section that appeared to directly answer the study topic. Before continuing with the remainder of the transcript, codes were compared, debated and adjusted. Transcripts were analysed in a joint coding meeting where the coding was compared. Both coders applied a code to a section of text (the second researcher re-read the section and re-considered if the code should be applied). New codes were generated as the researcher progressed through them. The codes were reviewed, compiled into an initial topic and sorted into bigger themes that appeared to answer the study question specifically. The aim of the study was to determine what each topic was about, what it was expressing and how it related to other themes. This was accomplished by constructing a final thematic map that demonstrated the relationship between themes (Maguire & Delahunt 2017). All analysed results were interpreted, backed and justified by relevant literature and consensus-seeking discussions with co-facilitator who was having more than 20 years of experience in psychiatric nursing and research and therefore an expert in that field. Data were kept in a locked area that only the researcher and supervisor could access. The researcher searched for themes, reviewed and defined them and wrote them up (Table 3).

**TABLE 3:** Themes.

Positive themes	Sub-themes
1. Psychological strategy	Individual counselling Therapeutic communication De-escalation technique
2. Physical strategy	Aggressive behaviour strategy Therapeutic environment
3. Chemical strategy	Administration of medications
4. Empowerment strategy	Training of staff Buy-in of management
5. Policy strategy	Guidelines Policy or acts

*Source:* Maguire, M. & Delahunt, B., 2017, ‘Doing a thematic analysis: A practical, step-by- step guide for learning and teaching scholars’, *Journal of all Ireland Journal of Higher Education* 9, 3352

### Ethical considerations

All individual participants included in the study gave informed consent. The Ministry of Health in Lesotho (ID113-2020) and University of Kwa-Zulu Natal (HSSREC/00002001/2020) granted ethical clearance for the study. All procedures were in accordance with the ethical principles as stated by the Belmont report, namely principles of beneficence, human dignity and justice.

## Results

### Demographic profile of participants

### Theme 1: Psychological intervention strategy of managing aggressive patient

It was evident from the data that the participants revealed a psychological intervention strategy of managing aggressive psychiatric patients. Participants reflected a psychological intervention strategy of managing aggressive psychiatric patient, which includes individual counselling, therapeutic communication and de-escalation techniques.

#### Sub-theme 1.1.: Individual counselling

All participants experienced this type of management strategy. Individual counselling was reflected as one-to-one communication that helped aggressive psychiatric patients discover their potentials:

‘Alright, we have what is called the counselling ehh [*pause*] in counselling, that is where you sit down with the patient, discuss with the patient, help the patient identify his or her problems and find the suitable way to solve his problems in the way that suits the patient. I think that is when we apply the mechanism but if the patient is still not in the, not mentally stable, I think that can wait yah [*pause*] that can wait until the patient is settled, yah [*pause*].’ (Individual Interview, Participant 1, Male, 25 years of experience)

This is in line with Haikal et al. (2023) who stated that participants viewed individual counselling as direct contact between the counsellor who is equipped with knowledge and skills and the client who gains self-awareness, understanding of present and future situations. Psychological intervention strategies were defined as relationships aimed at promoting a better adaption of the individual to a given situation and thereby optimising his or her personal resources in relation to autonomy, self-knowledge and self-help (Ricou et al. 2019).

#### Sub-theme 1.2: Therapeutic communication

Mental healthcare workers viewed therapeutic communication as giving and receiving information involving the sender and receiver of the information. Most of the mental healthcare workers experienced therapeutic communication as one reported:

‘I will communicate, I will try to communicate with the patient, like to calm the patient down ehh [*pause*] with the calm tone and with respect.’ (Individual Interview, Participant 7, Female, 22 years of experience)

This is congruent with Xue and Heffernan (2021) who reported that therapeutic communication is the nurse–patient relationship, which includes information exchange between a nurse and patient, based on shared respect and commitment in handling health issues of concern to the patient.

#### Sub-theme 1.3: De-escalation techniques

The majority of mental healthcare workers would use de-escalation techniques when the psychiatric patient became aggressive or violent, which are techniques that include communication and safety maintenance to reduce aggression or violence:

‘I set non-confrontational limit with the patient. This are strategies that I use on verbally aggressive patient.’ (Individual Interview, Participant 2, Male, 2 years of experience)

Slaato et al. (2021) asserted that de-escalation techniques frequently comprise teaching effective communication and active-listening skills, in addition to role-playing and includes practising the use of the preferred skills. Furthermore, Goodman et al. (2020) indicated that de-escalation techniques aim to decrease violence and aggression, by conveying a mutually agreeable resolution to the aggressor’s concerns.

### Theme 2: Physical strategy

For the majority of mental healthcare workers in the interview, physical strategy was viewed as management strategy of aggression and violence. Participants viewed physical strategy comprising of aggressive behaviour strategy and therapeutic environment.

#### Sub-theme 2.1: Aggressive behaviour strategy

Mental healthcare workers felt that aggressive behaviour strategy is a physical strategy used to detect weapons when assessing the patients’ wards to provide a safe and quiet area:

‘However, we do come across of ehh [*pause*] aggressive patients in the general wards because that is where we try to detect and remove any equipment that they can use to, to fight us, and this is where they start before they can be referred to psychiatric hospital, you may find that we come across patients who are, who have that physical aggression, sexual aggression and verbal aggression.’ (Individual Interview, Participant 8, Male, 6 years of experience)

Similarly, Richardson et al. (2019) indicated that measurement tools, risk assessment scales and pre-emptive identification and scoring systems have been established to allow prompt acknowledgement and calming prior to the escalation of aggression. Participants felt that environmental risk management interventions, such as reducing access to weapons, ensuring that items used in the provision of care are secured and cannot be utilised as weapons, are crucial (Victoria 2017)

#### Sub-theme 2.2: Therapeutic environment

According to the mental healthcare workers, a therapeutic environment has a considerable impact on the management of aggression and violence as it provides psychiatric patients with an opportunity to interact with mental healthcare workers and other patients, and it is where aggressive or violent psychiatric patients make decisions in their management and care of other patients as he or she becomes less dependent and less passive:

‘I try to make sure that the patient feels comfortable and try to provide safe environment so that he or she can calm down and have open communication with me.’ (Individual Interview, Participant 3, Male, 11 years of experience)

Consequently, Uys and Middleton (2015) affirmed that therapeutic environment promotes individuality of patients, accepts the humanity of patients by ensuring privacy and giving them living standards equal to those of general patients and also accepts the fact that patients possess a considerable degree of responsibility.

### Theme 3: Chemical strategy

Chemical strategy is viewed as crucial in managing aggressive or violent psychiatric patients as it utilises drugs and therefore useful as it calms and de-escalates aggression and violence.

#### Sub-theme 3.1 Administration of medications

The majority of mental healthcare workers indicated that medication administration is one of the most critical management strategies in providing care to aggressive and violent patients. Furthermore, they viewed medication administration as the process that includes preparing, checking, monitoring the effects of medications and teaching patients about medications so that their psychiatric disorders cannot trigger aggressive violence:

‘If the measures are not helpful, we prescribe medications. Here in this hospital, we normally use injections such as haloperidol 5mg stat and the [*pause*] we prescribe diazepam 10mg IM or sometimes we give 5mg IV to the patient. After administration, most patients calm down, some might fall asleep.’ (Focus group interview 3, Participant A, Male, 33 years of experience)

Roppolo et al. (2020) detailed that medications are administered to aggressive psychiatric patients to calm the patient without over-sedation, if non-pharmacological measures are not successful and are necessary whenever physical restraint of the patient is required to prevent injuries and complications associated with resisting restraint.

### Theme 4: Empowerment strategy

Mental healthcare workers talked a lot about empowerment as having a positive impact on both mental healthcare workers and aggressive or violent psychiatric patients. Participants viewed empowerment strategy comprising training of staff and buy-in of management.

#### Sub-theme 4.1: Training of staff

Mental healthcare workers indicated that education plays an important role in achieving organisational goals through a combination of organisational and workforce interests and is an essential factor contributing to their efficiency and organisation:

‘Yes, madam, I will like to be capacitated with physical break away techniques whereby we will be helped to, to have skills on what to do when we deal with aggressive patient like providing space between me and my patients, being able to or be next to exit door, trying by all means to, to protect my essential parts.’ (Focus group Interview 4, Participant A, Male, 10 years of experience)

According to Shams and Hattingh (2020), mental healthcare workers’ involvement in training leads to improved knowledge, skills and confidence to assist a mentally ill patient and decreases stigmatising attitudes that can lead to aggression. Empowerment as a key strategy for improving professional engagement and role development in hospital settings was unfolded as crucial (Nursalam et al. 2018).

#### Sub-theme 4.2: Buy-in from management or government

According to the views of mental healthcare workers, leadership is important in the management of aggression and violence, as it affects their retention. Management or the government would assist them by providing equipment to use in the clinical facility. The government or the management would assist with planning of the activities in the management of aggressive psychiatric patient and will also control activities during the management of aggressive behavior of psychiatric patients as to support the mental healthcare workers:

‘I think [*deep breathing*] you come to work [*pause*] our management has to see to it that we have every resource that can enable us to help us.’ (Individual Interview, Participant 4, Female, 4 years of experience)

Leadership has been demonstrated as crucial for creating a supportive work environment for nurses through which they are able to produce quality care and improve patient’s outcomes (Boamah et al. 2018). In addition, Zaghini et al. (2020) showed that patient satisfaction with apparent care was prejudiced by nurses’ actions and depersonalisation, which in turn were related to organisational context variables, including the quality of leadership.

### Theme 5: Policy strategy

According to the views of mental healthcare workers, policy is management strategy used in hospital setting to manage aggressive and violent psychiatric patients.

#### Sub-theme 5.1: Guidelines

Mental healthcare workers indicated guidelines as strategies that aim to safeguard both mental healthcare workers, aggressive and violent psychiatric patients and other patients by helping to prevent aggression or violent situations:

‘Emm [*pause*] setting boundaries is all about emm [*pause*] setting guidelines, rules, and regulations on what the patient will or won’t do with clear conscience.’ (Individual Interview, Participant 2, Male, 2 years of experience)

This is consistent with NICE (2015) who conveyed that guidelines aim to safeguard both staff and people who use services by helping to prevent violent situations and providing guidance to manage them safely.

## Discussion

The present study was carried out on 33 mental healthcare workers (psychiatric nurses, medical doctors and nursing assistants). The majority of participants were females. This may be because of the fact that nursing is a profession dominated by women (Rathobei et al. 2021). The study revealed that more participants were nurses with Diploma in general nursing and midwifery and those who had Certificate in Nursing Assistants. These results are in agreement with Abd El-hei (2017) who reported that nurses were not forced to improve their educational levels. However, the results of the study are contrary to Abdelrazak (2014), who studied nurses’ perception and practice of utilising the therapeutic community among hospitalised patients and stated that half of the studied nurses were in secondary nursing schools. Concerning years of experience of working, the majority of participants had more than 10 years of experience. This corresponds to the study by Abd El-hie (2017) which revealed that nurses had experience of more than 5 years.

The participants shed light on the problems related to management strategies of aggressive behaviour of psychiatric patients. Participants revealed that psychological intervention could be carried out successfully using de-escalation tactics, individual counselling, and therapeutic communication. Individual counselling was perceived by participants as a caring profession that deals with the process of assisting people in discovering and developing their skills and finding personal and social fulfilment. This is in line with McAllister and McCrae (2017) who stated that service user’s perceptions suggest that there is need for staff–patient interactions for a relatively short period. Furthermore, Moreno-Poyato et al. (2018) reported that nurses and service users had better perceptions regarding individual therapy. This may be owing to the fact that individual counselling identifies aggressive psychiatric patient’s needs and therefore prevents aggression. This is consistent with Tripathy and Sahu’s (2020) assertion that a therapist uses their professional skills for dealing with troubled individuals when providing individual counselling.

Participants viewed that therapeutic communication is exchanging and receiving messages from the therapist in order to identify and treat each person’s unique difficulties. According to Baby, Gale and Swain (2019), therapeutic communication is a skill necessary to develop therapeutic relationships, identify disorders and provide effective therapies. The study’s findings are consistent with those of Goodman et al. (2020), who stated that participants reported that communication skills are important particularly, speaking in a calm and controlled manner, giving simple and direct instructions, maintaining a composed exterior with open and non- threatening body language. This is congruent with Xue and Heffernan (2021) who reported that participants viewed therapeutic communication as the nurse–patient relationship that includes information exchange between a nurse and patient, based on shared respect and commitment in handling health issues of concern to the patient. In addition, Lankeren et al.’s (2020) study conducted in the Netherlands revealed clear and calm communication to be imperative as such communication could help control escalations and manage the anger of patients but also motivates patients to take their medication.

When the psychiatric patient became hostile and violent, the majority of participants used de-escalation tactics. These approaches include communication, self-regulation, assessment, actions and safety maintenance to lessen aggressiveness and violence. Goodman et al. (2020) stated that participants valued patient-centered approach encompassed in de-escalation technique and highlighted that it is crucial for one patient to be given time and space to reduce patient’s aggression.

Participants perceived physical strategy as a method of controlling anger and violence that included risk management measures for the surroundings. This is echoed by Barr et al. (2019) who stated that participants viewed risk awareness and risk assessment crucial and also expressed the importance of balancing security and safety with ensuring equity of care.

Participants saw the implementation of an aggressive behaviour strategy as a management tool for identifying hazards and providing safety. This is in line with Morphet et al. (2018), who stated that aggressive behavior approach is a method employed for weapon detection, to assess patients rooms for safety and prevention of noise in patients’ rooms.

Participants believed that the therapeutic setting offered safety and opportunities for interpersonal connections, which facilitated interactions between staff members and patients. Furthermore, Abd El-hie¹, Mourad and El-Azzab (2017) indicated in their study that nurses felt that therapeutic environment is designed to give patients a chance to interact with staff and other patients. It is also where patients make management decisions and take care of other patients as they become less dependent and passive.

The chemical method was described by participants as the management of patients using medications that can be utilised for severe behavioural problems in those with mental illnesses, with the intention of quickly relaxing and de-escalating potentially dangerous situations. The majority of participants said that one of the most important management strategies for treating aggressive and violent patients is medication administration. Similarly, participants in Muir Cochrane et al.’s (2020) study highlighted the importance of pharmacological restraint in managing aggressive and violent behaviour in an emergency. According to Costa et al. (2018), participants viewed medicine administration as one of the most important aspects of patient care in hospitals because it is the primary tool utilised to treat the patient’s illness. In addition, Härkänen et al. (2018) noted that the process of administering medications also entails the preparation and inspection of medications, observation of the effects of medications, instruction of patients regarding medications and updating of nurses’ own knowledge.

Mental healthcare professionals reported feeling empowered regarding the management strategies of aggressive behaviour of psychiatric patients. In the study conducted by Krull et al. (2019), participants’ self-reported changes in knowledge, skills, ability, confidence and preparedness demonstrated significance for a short-term change based on the pre- and postsurvey analysis. According to Nursalam et al. (2018), empowerment is a vital technique for enhancing engagement and role development in hospitals and is a tried-and-true method for fostering a healthy work environment in organisations. Through a combination of organisational and workforce interest, participants testified that education plays a significant role in accomplishing organisational goals and is a key contributor to their efficiency and organisation. This is consistent with the assertion made by Chaghari et al. (2017), that training is crucial to accomplishing organisational objectives. Furthermore, in the study conducted by Abd El-hie et al. (2017) that aimed to assess nurses’ knowledge and attitudes, participants felt that attending conferences and seminars updated their information regarding different treatment modalities and how to apply their knowledge in a practical field.

Participants felt that as it has an impact on their retention, leadership is crucial in the management of hostility and violence. Similarly, Barr et al. (2019) stated that participants felt employers had a responsibility to be selective when recruiting staff to work with aggressive psychiatric patients to ensure effective team work and develop a positive and safe workplace culture. Correspondingly, Morsiani, Bagnasco and Sasso (2017) results viewed that leadership is the capacity to direct, inspire and drive others towards the accomplishment of a predetermined objective and the continuous pursuit of good leadership.

Participants described policy as a management technique used in hospitals to control violent and aggressive psychiatric patients because it protects mental healthcare professionals, visitors and other patients in addition to controlling violent and aggressive psychiatric patients. This is congruent with the study conducted by Yousefinezhadi et al. (2017), where participants claimed that at the national level, the Ministry of Health is responsible for formulating policies, allocating funds, organising, supervising, coordinating and evaluating the healthcare services offered by a variety of public and private healthcare organisations and institutions.

Participants learned about policies as a management method intended to safeguard patients, visitors, aggressive and violent patients and mental health professionals. According to Health (2015), recommendations aim to protect both staff and clients by assisting in the prevention of violent situations and offering advice on how to appropriately handle them when they do arise. Baby et al. (2018) reported that participants felt professional and governing bodies have developed guidelines and recommendations to be covered in aggression prevention and management programmes. Additionally, the study by Sim, Ahn and Hwang (2020) pointed out that to safeguard nurses from external and interpersonal concerns while upholding an ethical attitude and paying attention to patients’ legal rights, laws surrounding general clinical safety must be strengthened. Moreover, the study by Ferracuti et al. (2022) revealed that a lack of violence at any level, staff training with clear objectives, a thorough and complete investigation of workplace violence occurrences and improved communication between health professionals, patients and their families are all necessary for the safety of health workers.

### Limitations of the study

The study took place in Maseru district only and did not include other districts.

## Recommendations

Further research is necessary, including other districts of Lesotho, using both qualitative and quantitative research approaches. Another recommendation is the capacitating of mental healthcare workers through in-service training to enhance their management strategies of aggressive and violent behaviour by psychiatric patients.

## Conclusion

The mental healthcare workers’ experiences in managing aggression and violent behaviour of psychiatric patients were an expression of psychological intervention, physical, chemical, empowerment and policy strategies. The management strategies were evident among all mental healthcare workers who took part in this study.
